# Development and Optimization of Medical-Grade Multi-Functional Polyamide 12-Cuprous Oxide Nanocomposites with Superior Mechanical and Antibacterial Properties for Cost-Effective 3D Printing

**DOI:** 10.3390/nano12030534

**Published:** 2022-02-04

**Authors:** Nectarios Vidakis, Markos Petousis, Nikolaos Michailidis, Sotirios Grammatikos, Constantine N. David, Nikolaos Mountakis, Apostolos Argyros, Orsa Boura

**Affiliations:** 1 Mechanical Engineering Department, Hellenic Mediterranean University, 71004 Heraklion, Crete, Greece; vidakis@hmu.gr (N.V.); markospetousis@hmu.gr (M.P.); mh90@edu.hmu.gr (N.M.); 2 Physical Metallurgy Laboratory, Mechanical Engineering Department, School of Engineering, Aristotle University of Thessaloniki, 54124 Thessaloniki, Macedonia, Greece; nmichail@auth.gr (N.M.); aposargy1@ee.duth.gr (A.A.); 3 Centre for Research & Development of Advanced Materials (CERDAM), Center for Interdisciplinary Research and Innovation, Balkan Center, 57001 Thessaloniki, Macedonia, Greece; 4 Group of Sustainable Composites, Department of Manufacturing and Civil Engineering, Norwegian University of Science and Technology, 2815 Gjøvik, Norway; orsa.boura@ntnu.no; 5 Manufacturing Technology & Production Systems Laboratory, School of Engineering, International Hellenic University (Serres Campus), 62124 Serres, Macedonia, Greece; david@ihu.gr

**Keywords:** three-dimensional (3D) printing, nanocomposites, polyamide 12 (PA12), cuprous oxide (Cu_2_O), fused filament fabrication (FFF), biocidal efficiency, mechanical properties

## Abstract

In the current study, nanocomposites of medical-grade polyamide 12 (PA12) with incorporated copper (I) oxide (cuprous oxide-Cu_2_O) were prepared and fully characterized for their mechanical, thermal, and antibacterial properties. The investigation was performed on specimens manufactured by fused filament fabrication (FFF) and aimed to produce multi-purpose geometrically complex nanocomposite materials that could be employed in medical, food, and other sectors. Tensile, flexural, impact and Vickers microhardness measurements were conducted on the 3D-printed specimens. The fractographic inspection was conducted utilizing scanning electron microscopy (SEM), to determine the fracture mechanism and qualitatively evaluate the process. Moreover, the thermal properties were determined by thermogravimetric analysis (D/TGA). Finally, their antibacterial performance was assessed through a screening method of well agar diffusion. The results demonstrate that the overall optimum performance was achieved for the nanocomposites with 2.0 wt.% loading, while 0.5 wt.% to 4.0 wt.% loading was concluded to have discrete improvements of either the mechanical, the thermal, or the antibacterial performance.

## 1. Introduction

A new era in manufacturing processes is met through the utilization of additive manufacturing (AM), which is currently considered as the most prominent manufacturing method in a wide range of industrial and research applications [1,2,3,4,5,6]. Developments and new implementation methods are presented at a geometrically increasing rate in automotive/aerospace industries [2,7], building and constructions [8,9], electronic devices [10,11], medical devices [7,12], and many others. The high interest for AM in academia and industry is attributed to the capabilities given to users from this manufacturing method [8,13]. Manufacturing extremely complex to manufacture geometries and low operational costs are some of the benefits stemming from the utilization of 3D printing [3,14] at a minimum percentage of wastage. Even if wastages are produced, they are, to a high extent, recyclable under certain circumstances [7,15].

In fused filament fabrication (FFF), which belongs to the material extrusion (MEX) family of the AM technologies [16,17,18], raw materials are mainly thermoplastic polymers and/or composites [19,20,21,22,23,24], with their mechanical properties thoroughly investigated in most cases [25,26,27]. The form of the raw material is in filament form and usually has a diameter of 1.75 mm. A radical increase in the range of filaments available in FFF is achieved through research in material science [8,28,29,30]. In this fashion, many composite materials are presently available in the market in a filament form suitable for FFF, which exhibits improved performance in terms of mechanical, electrical, thermal, antibacterial, and other properties, over the conventional ones.

As in other AM technologies, the operational principle is based on a layer-by-layer material deposition. The material’s fusion is achieved at high temperatures by using a hot end positioned inside an electronically driven head. This “3D printing” head can move in the X and Y direction. A repeated process of material deposition at specific paths in a layer-wise fashion creates the final part.

Three-dimensional printing limitations are mainly due to the operational principle of layer-by-layer manufacturing [31,32]. This fabrication procedure often results in poor adhesion between the 3D printing layers, while many unpredicted parameters or minor faulty settings may cause voids and deficiencies (which in many cases are not visible by the naked eye) in the parts. Such manufacturing complications may impact applications, such as in the medical industry, in which “tiny” voids may favor bacterial growth. Similar problems exist in food industry applications [33,34], while the mechanical and thermal performance of the parts are also affected. To overcome these issues and improve the performance of the parts produced by the FFF technology, composite materials are developed using well-established polymers in engineering applications (usually called engineering-grade polymers) as the matrix. Such composites are fabricated using fillers in micro/nano or fiber form to achieve improved mechanical [12,35,36], thermal [37,38,39], or electrical performance [11,40,41], compared to the matrix alone. Many additives hinder the flow of the material, so several studies on the flowability of composites exist in this field [12,42]. Generally, research is targeted at the creation of multi-purpose materials to achieve optimum performance with the addition of the filler to the polymer matrix.

Polyamides are well-known polymers and are widely used in engineering applications. A polyamide used so far, mainly in selective laser sintering (SLS) and binder jet technologies of AM, is polyamide 12 (PA12) [29,43] PA12 is a material that can be employed in many implementations due to its thermomechanical properties. It is not widely used in MEX implementations yet, with its filament market share being significantly lower than other materials [44]. Still, it was studied in literature and used as a pure material and as a matrix material in composites in several important MEX 3D printing applications [31,35,45,46,47,48,49,50] and as a matrix material with metals and oxides as fillers in AM applications requiring antibacterial properties from the materials [51,52,53,54]. Its enhanced toughness properties and its ability to widely extend before it breaks are some of its merits for utilization in 3D printing [11,13,29,38], while its flow behavior is trouble-free for both the extrusion and the 3D printing process [37]. Additionally, it has very good behavior when mixed with additives for the improvement of its properties [38,55]. Finally, its performance does not seem to degrade even after five (5) recycling loops [37]. As a result, PA12 has a high potential as a matrix material for the development of multi-purpose filaments in the ΜΕΧ technology.

Copper (I) oxide (cuprous oxide), is met with the chemical formula of Cu_2_O. It is an inorganic compound with a cubic crystal structure [56] and is utilized in a wide range of applications, such as semiconductors [41,57], medical applications [58,59,60], and others. Copper (I) oxide’s antibacterial performance has been studied and it is well documented in the literature [61,62]. Copper (Cu) and copper oxides have been used as fillers in vat photopolymerization to induce antibacterial performance to polymeric resins [63,64]; however, no studies exist yet that are related to ΜΕΧ 3D printing technology [65,66,67,68]. Hence, they were selected in this study as potential fillers to introduce multifunctional behavior in the prepared nanocomposite materials. Multifunctionality, which is attributed to the effect of the filler on the matrix material performance, could be the main advantage for applications where a wide range of properties are necessary, i.e., in medical devices, and consequently, multiple materials and a wide range of manufacturing methods have been utilized so far [7]. The development of such multifunctional composites for 3D printing creates foundations of future implementation in such applications, while a further potential for more complex geometry fabrication and cost reduction still exists.

In this study, PA12-Cu_2_O nanocomposites were prepared at various ratios, to investigate the effect of cuprous oxide loading on the properties of the nanocomposite material. Weight-to-weight loadings of 0.5 wt.%, 1.0 wt.%, 2.0 wt.%, 4.0 wt.%, and 6.0 wt.% were selected for the study. A thermomechanical melt mixing process was employed, and specimens were fabricated, with FFF 3D printing employing the produced filaments. Their mechanical, thermal, and antibacterial properties were investigated to fully characterize the prepared nanocomposites, which presented enhanced properties in all tests conducted compared to the matrix. The 2 wt.% nanocomposite had the optimum performance overall, proving the multifunctional characteristics of the material. It is worth noting that an 8.0 wt.% Cu_2_O nanocomposite was also prepared, but it exhibited flow issues during the extrusion process, so it was not further considered, demonstrating that for specimens prepared with the methodology followed herein there is an upper threshold in the concentration of the filler in the specific matrix.

## 2. Materials and Methods

**Figure 1:**
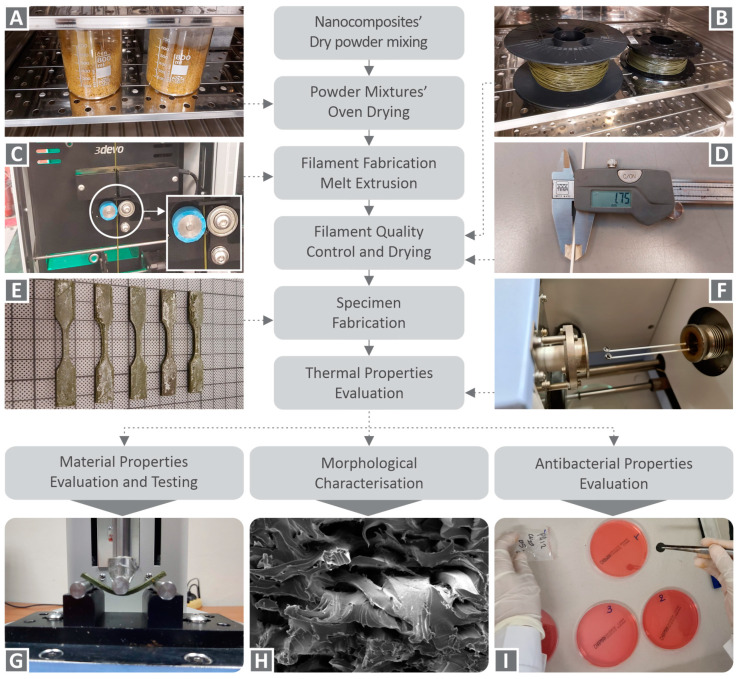
A flow chart with the steps of the process followed in the current study.

Figure 1 summarizes the procedures followed in the current study.

### 2.1. Materials

Medical grade polyamide 12 (PA12) thermoplastic material was procured from Arkema (Colombes, France) of Rilsamid PA12 AESNO TL grade. The matrix material was in the form of fine granules with the following characteristics, according to the manufacturer’s technical data sheet: a density of 1.01 g/cm^3^ (ISO 1183), melt volume–flow rate (MVR) of 8.0 cm^3^/10 min (ISO 1133) at 235 °C/5.0 kg, Vicat softening temperature at 142 °C (ISO 306/B50) and melting temperature at 180 °C (ISO 11357-3). It should be mentioned that the current PA12 grade also has low-percentage additives for the improvement of its heat stabilization, lubrication, and UV stabilization. According to the PA12 material manufacturer, this specific grade should not be implanted in the body or be in contact with bodily fluids or tissues for a time period greater than 30 days. Cuprous oxide (Cu_2_O) filler was procured from Nanografi Nanotechnology SA (Tallin, Estonia) in form of a nanopowder. More specifically, the particles have a diameter of 80 nm with a purity of 99.5%.

### 2.2. Filament and Specimens’ Fabrication

An extrusion process was followed to fabricate the necessary filaments for the current study. For the extrusion process a single-screw extruder was employed, specifically a 3D Evo 450 Composer (3D Evo B.V., Utrecht, the Netherlands). This machine is equipped with a screw with geometry specially designed for materials and mixing nano additives, according to the manufacturer. It also features a four-zone heating barrel, a built-in winding system, and an optical sensor (which automatically controls the winding rotational speed) to secure real-time monitoring of the produced filament diameter (1.75 mm). Before the extrusion process, the material of the composite matrix was dried at 80 °C for 24 h. The filler and matrix materials were mixed using dry mixing in a high rotational speed blend cutter for half an hour to produce the best possible homogenized mixture at room temperature. Then, the mixed materials were further dried for 4 h at the same temperature and poured into the extruder’s hopper for filament production. The produced filament was shredded in a 3devo laboratory shredder (3D Evo B.V., Utrecht, the Netherlands) and the pellets were then fed again to the extruder for the final step of the filament production process. The extrusion settings applied for the filament production of the examined matrix and nanocomposite materials are 185 °C at heating zone 4 (closer to the hopper induction), 220 °C at intermediate heating zones 2 and 3, and 210 °C at heating zone 1 (closer to the nozzle). Built-in cooling fans of the extruder were set to 50% capacity and the screw’s rotational speed was set to 8.5 rpm. An average deviation in the diameter of the filament of 0.08 mm from the goal value of 1.75 mm was measured.

**Figure 2:**
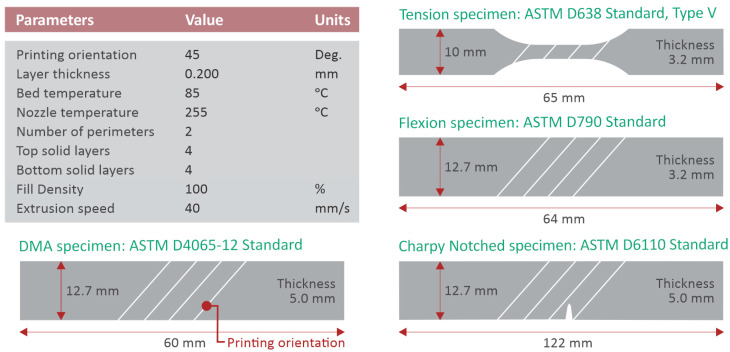
Fundamental fused filament fabrication 3D printing settings applied during specimen manufacturing.

A Craftbot Plus (Craftbot Ltd., Budapest, Hungary) FFF 3D printer was employed for the specimens’ fabrication. Craftbot was equipped with an all-metal hot-end and a nozzle of 0.4 mm diameter. The 3D printer’s bed is made from an aluminum sheet, and on this surface, a polyetherimide (PEI) printing surface was fitted to improve the adhesion of the first layer to the bed. Figure 2 shows the fundamental 3D printing parameters set through the Craftware slicer software tool, which was utilized for the g-code preparation. It should be also noted that the fans of the nozzle were switched off during the entire 3D printing process. Furthermore, all other parameters that are not referred to in Figure 2 or described above were set to the default values that apply for the case of PA material of the Craftware software tool. All printing settings were kept constant for all the examined nanocomposite cases.

### 2.3. Characterization and Testing

Tensile tests were performed following the ASTM D638-02a standard. A type V specimen with 3.2 mm thickness was prepared for the tests. An Imada MX2 (Imada Inc., Northbrook, IL, USA) laboratory tensile testing machine was employed, with the elongation speed at the standardized grippers set to 10 mm/min according to the ASTM standard. Flexural tests were conducted following the ASTM D790 standard on the same device, applying a three-point bending setup (52 mm span), with the bending speed set to 10 mm/min. Both tensile and bending tests were conducted at a room temperature of 21 °C. Charpy impact tests were also conducted on notched specimens, following the ASTM D6110 standard. For the impact tests, a Terco MT 220 (Terco AB, Kungens Kurva, Sweden) apparatus was utilized. The release height of the apparatus’ hammer was kept constant at 367 mm for all tests. In these three mechanical tests, six specimens were manufactured and tested for each different material in each test.

Vickers microhardness measurements were conducted on the surface of tensile specimens after grinding them to achieve a smooth surface. The microhardness measurements are related to the material’s mechanical response [69] and were taken using an Innova Test 300 apparatus (Innovatest Europe BV, Maastricht, the Netherlands). The indentations’ duration was set to 10 sec, and the applied load was 200 gF.

Scanning electron microscopy (SEM) was facilitated through a JEOL JSM 6362LV (Jeol Ltd., Peabody, MA, USA) electron microscope in high-vacuum mode at 20 kV acceleration voltage on sputtered-gold coated specimens. Atomic force microscopy (AFM) measurements were performed on the fabricated filaments with the aid of a scanning probe microscope Microscope Solver P47H Pro (NT-MDT, Moscow, Russia). AFM images were captured under a room temperature of 21 °C and resonance frequency of 300 kHz. Moreover, roughness measurements were taken on the specimens’ surface with a Contour GT (Bruker Nano GmbH, Berlin, Germany) laboratory machine.

A thermogravimetric (TGA) analysis was conducted on samples of approximately 10 mg, and taken from the 3D-printed specimens, using a Perkin Elmer Diamond TGA/DTGA (Waltham, MA, USA) laboratory equipment. Tests were conducted in the temperature range of 40 °C to 550 °C with a temperature ramp of 10 °C/min.

Finally, the antibacterial efficacy of the nanocomposites was assessed against gram-positive Staphylococcus aureus (*S. aureus*) and gram-negative Escherichia Coli (*E. Coli*) bacteria strains. PA12/Cu_2_O nanocomposites specimens of cylindrical form with a 12 mm diameter and a height of 5 mm were tested according to the agar diffusion model [70] in a microbiological laboratory with equipment properly sterilized before usage. Petri dishes with an 85 mm diameter and suitable bacterium growth agent (MC.2, C.010066 for the *E. Coli* and Chapman, C.010068 for *S. Aureus*) were employed, with the developed inhibition zones (IZ) measured using optical equipment after 24 h at 37 °C.

## 3. Results

### 3.1. Mechanical Behavior

**Figure 3:**
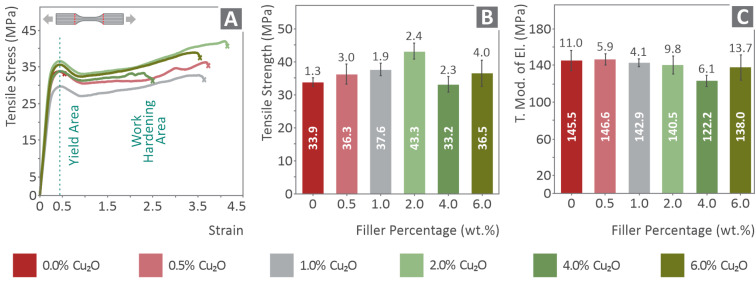
(**A**) Typical tensile stress–strain diagrams, (**B**) average tensile strength at break (MPa) vs. the concentration of the fillers, and (**C**) average tensile modulus of elasticity (MPa) vs. the concentration of the fillers for all tested materials. The numbers inside the bars are the average calculated values for each case, while the numbers close to the error bars are the corresponding calculated deviations.

In Figure 3A, typical tensile-derived stress (MPa)–strain (mm/mm) curves are shown for each of the tested materials. The outcome of these tests is summarized in the bar charts of Figure 3B,C. Τhe average tensile stress at break and the average calculated tensile modulus of elasticity (MPa) are shown. The addition of cuprous oxide in the PA12 polymer matrix results in a strengthening effect that augments with the increase of the fillers loading, although the modulus of elasticity oscillates around a constant value, remaining practically unaffected by the cuprous oxide concentration. The tensile strength is continuously increasing for the filler’s loadings up to 2.0 wt.%, where the maximum value is exhibited (approximately 28% increase compared to the pure PA12).

**Figure 4:**
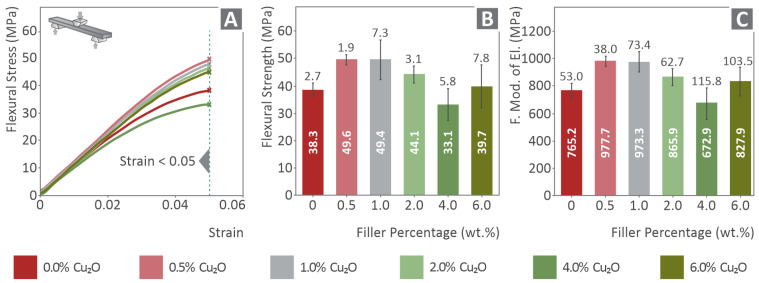
(**A**) Typical flexural stress versus strain, (**B**) average flexural strength at 5% strain versus filler’s concentration and, (**C**) average flexural modulus of elasticity versus filler’s concentration in all the examined cases. The numbers inside the bars are the average calculated values for each case, while the numbers close to the error bars are the corresponding calculated deviations.

In Figure 4A, typical flexural stress–strain (mm/mm) curves are shown for each of the examined material variations. Flexural stress and strain values were calculated according to the corresponding equations provided by the ASTM D790 international standard followed in this work. The average flexural stress at the strain of 5% (according to the standard instructions, as no brake occurred on the specimens) is shown in Figure 4B in comparison to the filler’s loading. In Figure 4C, a comparison of the calculated flexural modulus of elasticity is presented for each material tested. An increase in the flexural strength was observed for the filler’s loading up to 1.0 wt.%. The flexural modulus of elasticity is calculated about 27% higher than the corresponding value of pure PA12 (matrix) when loading with filler concentrations of 0.5 wt.% and 1.0 wt.%. A slight decrease of the flexural modulus of elasticity was observed at 2.0 wt.% cuprous oxide nanocomposite, but it is still higher than the value of the matrix. The average flexural modulus of the 2 wt.% loading nanocomposite is about 13% higher than the pure PA12, but, considering the deviations in these values, the difference between these two materials is small.

**Figure 5:**
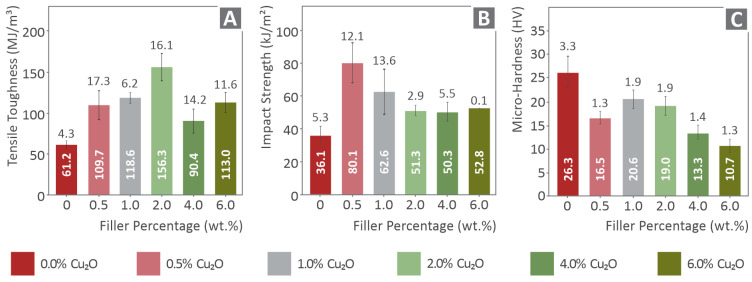
(**A**) Calculated average toughness (MJ/m^3^) vs. filler’s percentage (wt.%) comparison, (**B**) average impact strength (kJ/m^2^) vs. cuprous oxide (wt.%) ratio comparison, (**C**) average measured Vickers microhardness (HV) vs. filler’s ratio (wt.%) comparison.

In Figure 5A, the calculated toughness (MJ/m^3^) is presented for all tested materials. Toughness is a calculated measure provided as an integral of the tensile stress to strain curve. Generally, the toughness measure is related to the absorbed energy during the tension of the specimens. A higher toughness value could result in a more “fail-safe” mechanism, as the integral of the stress–strain curve is calculated up to the breaking point during the experiment. A steep increase in toughness was observed at 2.0 wt.% filler’s concentration (almost 255% increase), while all PA12/Cu_2_O nanocomposites have higher toughness values compared to the pure PA12. In Figure 5B, the average impact strength (kJ/m^2^) is shown for each material tested during the current study. The addition of cuprous oxide in the matrix material affects the material’s impact performance. PA12/Cu_2_O 0.5 wt.% exhibited the highest increase rate, of approximately 220% when compared to the pure matrix, while all other filler’s loadings exhibit almost a 100% increase compared to neat PA12. In Figure 5C, Vickers microhardness average measurements are shown for all nanocomposites and the pure material. The nanocomposites’ microhardness tends to decrease with the increase of the filler’s concentration and the maximum decrease was observed for PA12/Cu_2_O 6.0 wt.% (approximately 150% decrease when compared to pure PA12).

### 3.2. Morphological Analysis

**Figure 6:**
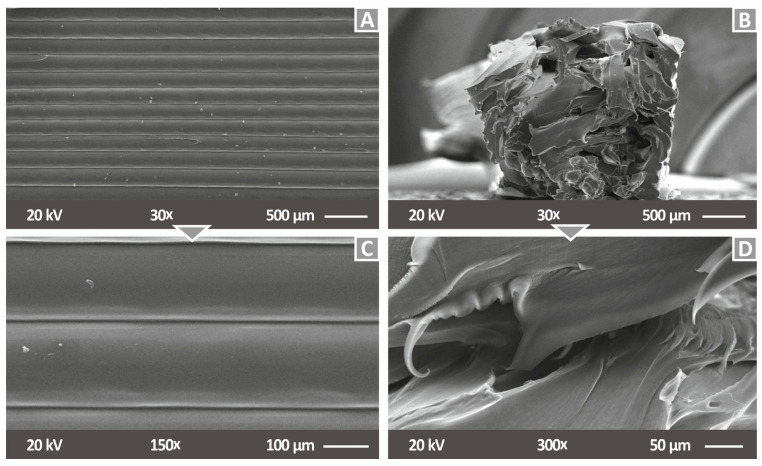
SEM images of tensile tests specimens build with pure PA12 material in this work: (**A**) side surface at 30× magnification, (**B**) fracture surface at 30× magnification, (**C**) side surface at 150× magnification, and (**D**) fracture surface at 150× magnification.

**Figure 7:**
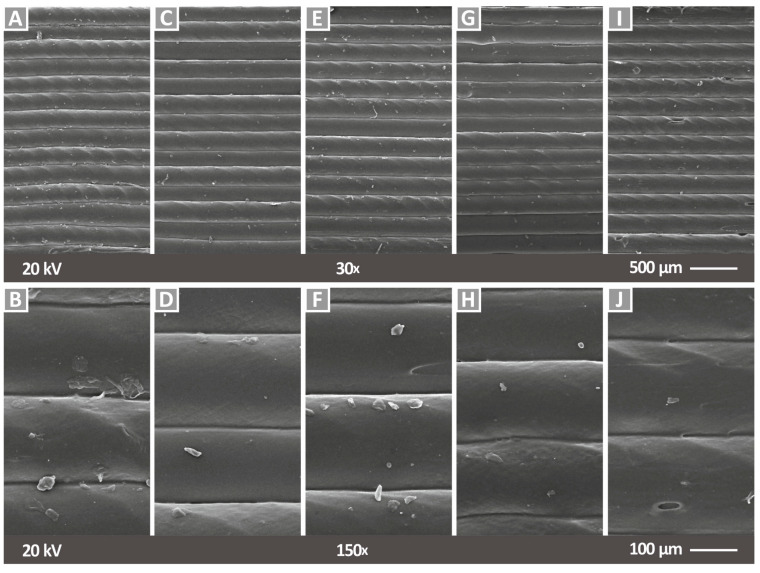
SEM images of the side surface of flat specimens (the building direction is upwards): (**A**) PA12/Cu_2_O 0.5 wt.% at 30×, (**B**) PA12/Cu_2_O 0.5 wt.% at 150×, (**C**) PA12/Cu_2_O 1.0 wt.% at 30×, (**D**) PA12/Cu_2_O 1.0 wt.% at 150×, (**E**) PA12/Cu_2_O 2.0 wt.% at 30×, (**F**) PA12/Cu_2_O 2.0 wt.% at 150×, (**G**) PA12/Cu_2_O 4.0 wt.% at 30×, (**H**) PA12/Cu_2_O 4.0 wt.% at 150×, (**I**) PA12/Cu_2_O 6.0 wt.% at 30×, and (**J**) PA12/Cu_2_O 6.0 wt.% at 150×.

In Figure 6, SEM images from the side of tensile specimens are shown for two (2) different magnifications for pure PA12 material (side surface—Figure 6A,C and fracture surface—Figure 6B,D). Figure 7 shows SEM images of the side of tensile specimens at all different loadings for the PA12/Cu_2_O nanocomposites produced in this work. Figure 7A,C,E,G,I presents the PA12/Cu_2_O 0.5 wt.% to PA12/Cu_2_O 6.0 wt.% nanocomposites at a magnification of 30×, while respective images are shown in Figure 7B,D,F,H,J in a higher magnification of 150×. Through these images, a good correlation of the produced layer height with the set one in the 3D printer (200 microns) can be observed for all tested materials. Moreover, a fine interlayer fusion is presented along the raster, indicating a fine 3D printing quality, while a slight twisting effect can be observed. This slight twist in the nanocomposites’ flow may have occurred from minor remnants in the 3D printer’s nozzle, but there is not any obvious impact on the experimental results and no flow rate change was noticed during the specimens’ fabrication. Specifically, for PA12/Cu_2_O 6.0 wt.%, shown in Figure 7I,J, some minor voids are observed, probably due to the high concentration of cuprous oxide, which may cause minor agglomeration effects. Small abnormalities shown in the filament strands in the side surface of the specimens can be attributed to 3D printing deficiencies and are not related to the additives in the materials, as similar abnormalities are also shown in the pure PA12 (Figure 6A,C). These are probably due to some remaining material in the nozzle. The combination of PA12/Cu_2_O 8.0 wt.% was not possible to be realized, as the flow of this material could not be achieved even during the filament extrusion process. The presence of minor voids at a concentration of the filler of 6.0 wt.% and a flow disability at 8.0 wt.% are strong indications of saturation in the fillers’ concentration.

**Figure 8:**
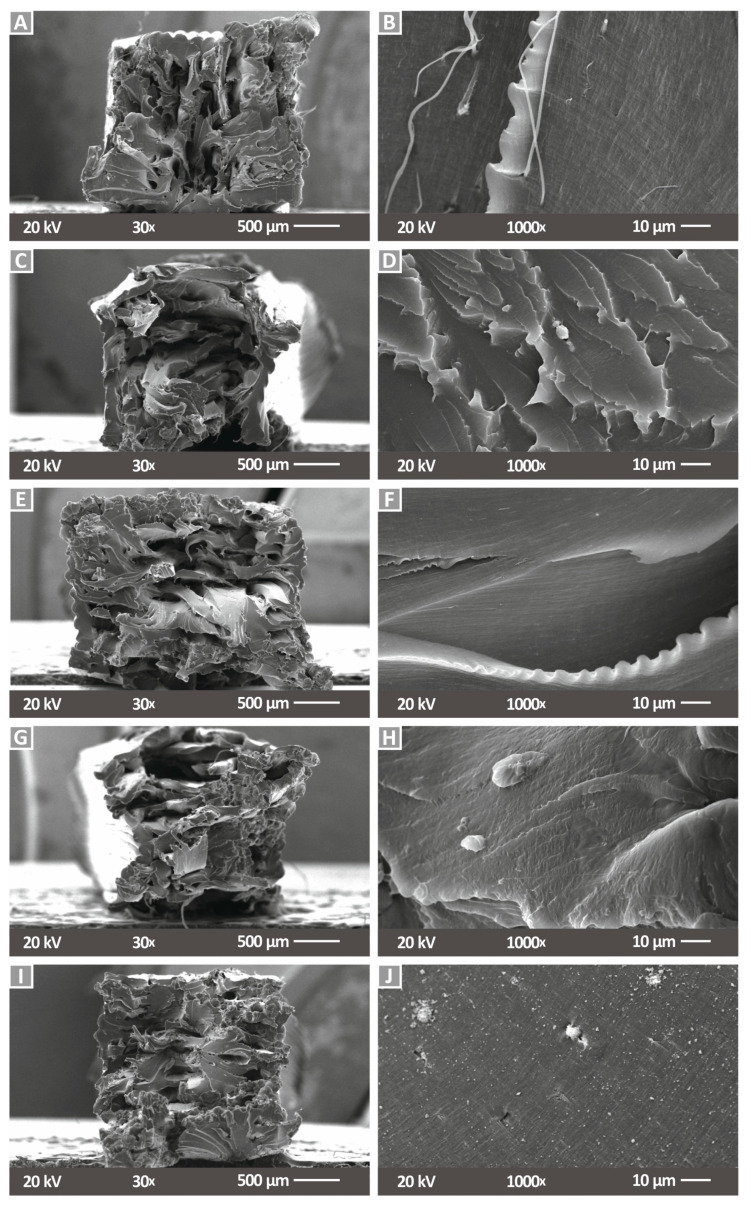
SEM images of tensile specimen fractured surfaces: (**A**) PA12/Cu_2_O 0.5 wt.% at 30×, (**B**) PA12/Cu_2_O 0.5 wt.% at 1000×, (**C**) PA12/Cu_2_O 1.0 wt.% at 30×, (**D**) PA12/Cu_2_O 1.0 wt.% at 1000×, (**E**) PA12/Cu_2_O 2.0 wt.% at 30×, (**F**) PA12/Cu_2_O 2.0 wt.% at 1000×, (**G**) PA12/Cu_2_O 4.0 wt.% at 30×, (**H**) PA12/Cu_2_O 4.0 wt.% at 1000×, (**I**) PA12/Cu_2_O 6.0 wt.% at 30×, and (**J**) PA12/Cu_2_O 6.0 wt.% at 1000×.

SEM images taken from the fractured area of tensile specimens for each tested nanocomposite material are shown in Figure 8, where Figure 8A,C,E,G,I corresponds to PA12/Cu_2_O 0.5 wt.% to PA12/Cu_2_O 6.0 wt.% at a magnification of 30×, and Figure 8B,D,F,H,J corresponds to a magnification of 1000×. Massive plastic deformation of the filament strands can be observed in the fracture images and any voids shown are attributed to the strands’ deformation during the failure of the specimens and not on the 3D printing flaws affecting the quality of the specimens. By observing the fractured areas, deformation zones and slight stiffening are evidenced. Necking is more intense at lower filler concentrations and is gradually descending with the increase of the cuprous oxide concentration. In Figure 8I, an almost “sharp” fractured area is shown, which implies a brittle failure. This tendency for a stiffer behavior in the nanocomposites as the filler’s loading increases can also be observed in higher magnifications. Sharp edges in a “wavy” pattern imply a gradual fracture mechanism, which is following the PA12 material’s plastic behavior. As the filler concentration increases, a reduction of these “waves” peaks is observed, which means that a more sudden break occurred. In nanocomposites with a cuprous oxide loading of 4.0 wt.% (Figure 8G,H) and 6.0 wt.% (Figure 8I,J), an almost uniform fractured area can be observed.

**Figure 9:**
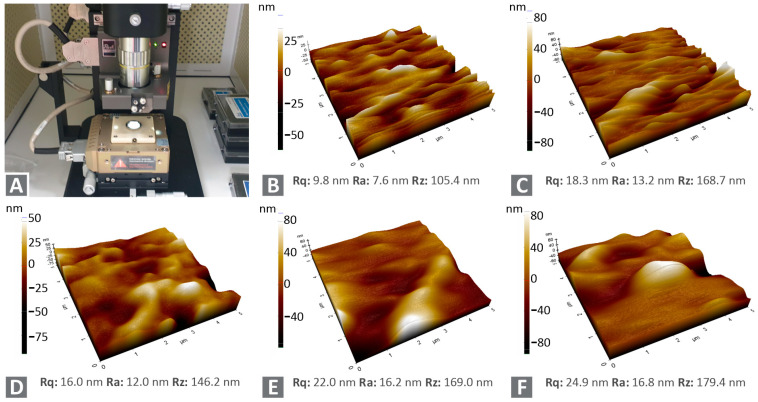
AFM measurements conducted to extruded filament of (**A**) Experimental setup, (**B**) PA12/Cu_2_O 0.5 wt.%, (**C**) PA12/Cu_2_O 1.0 wt.%, (**D**) PA12/Cu_2_O 2.0 wt.%, (**E**) PA12/Cu_2_O 4.0 wt.%, and (**F**) PA12/Cu_2_O 6.0 wt.%.

The filaments’ surface roughness was measured using atomic force microscopy (AFM). Figure 9 shows measurements conducted on the filaments’ surface for PA12/Cu_2_O 0.5 wt.% (Figure 9B) to PA12/Cu_2_O 6.0 wt.% (Figure 9E), respectively. The overall highest roughness values were measured at the filament’s surface with a concentration of 6.0 wt.%, revealing an Rz value (obtained from the AFM device for the measured 5 μm× 5 μm area) of 180 nm. Such an Rz value corresponds to fine surface quality. A tendency for the roughness of the filament to increase was obtained as the filler’s concentration increased, which was rather expected. The fine filament’s surface quality for all nanocomposites tested during the current study was confirmed through the AFM measurements, as the roughness measured exhibited values demonstrating that it is not expected to cause problems or modify the material’s flow during the 3D printing process.

**Figure 10:**
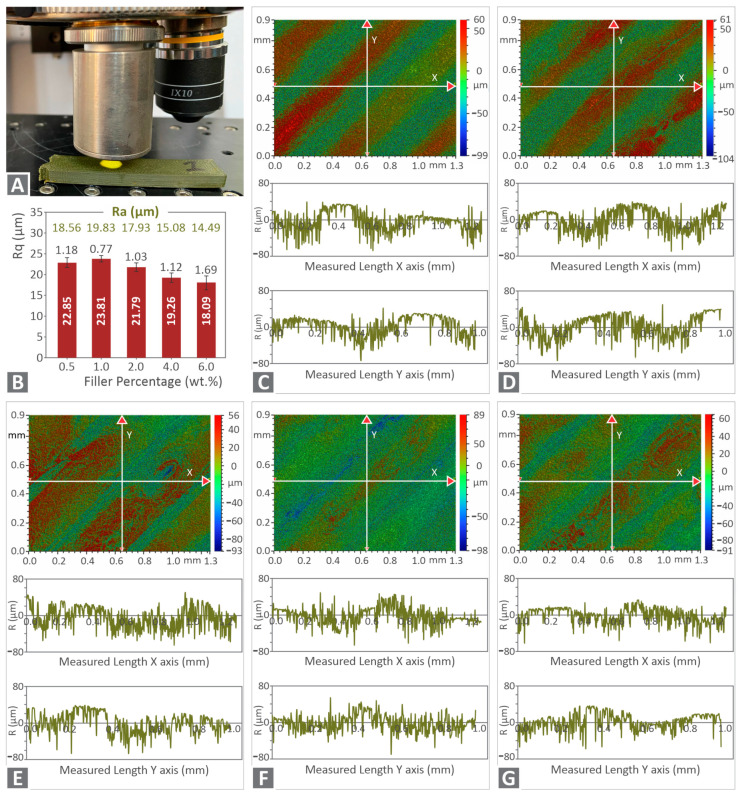
(**A**) Typical roughness measurements setup on the 3D-printed specimens, (**B**) average Rq (μm) calculated vs. the filler concentration (wt.%), along with their deviations (three measurements were taken in each specimen). Typical measurements conducted to the specimens’ area are presented in a graphical color map for the measured surface and a vectoring format for the X and the Y direction for (**C**) PA12/Cu_2_O 0.5 wt.%, (**D**) PA12/Cu_2_O 1.0 wt.%, (**E**) PA12/Cu_2_O 2.0 wt.%, (**F**) PA12/Cu_2_O 4.0 wt.%, and (**G**) PA12/Cu_2_O 6.0 wt.%.

Surface roughness measurements were also performed on the specimens’ 3D printed surface (see Figure 10). Figure 10A presents the measurements setup, while Figure 10B–G reveals the roughness measurements in various forms, i.e., as summary bar charts and corresponding deviations, profile plots, and surface scans for all examined nanocomposites. Overall, the increase of the filler loading decreases the roughness of the 3D-printed specimens. Mean roughness (either Rq or Ra) has minor differences for all tested cases in comparison to the specimens’ dimensions. This means that a fine 3D printing quality was achieved even at higher filler concentrations. Although the absolute measured roughness values slightly differ, a slight decrease in the surface roughness as the filler ratio increases was observed, indicating a fine dispersion of the filler in all nanocomposites. Low dispersion could probably cause agglomerations, which in turn would have resulted in a surface roughness increase. Filler agglomerations in the specimens’ surface could increase the surface roughness of the specimens. As the measured surface roughness is not significantly different between the cases studied, such agglomerations are not present at least on the surface of the specimens. Good surface roughness values indicate good deposition and cooling of the material specimens’ surfaces during the 3D printing process. Keeping constant flow rates for all materials and generally constant 3D printing settings could cause an increase in the pressure of the nozzle during the extrusion of the material and as a result, an increase in the “ironing” effect that could potentially contribute to such measurements.

### 3.3. Thermal Properties

**Figure 11:**
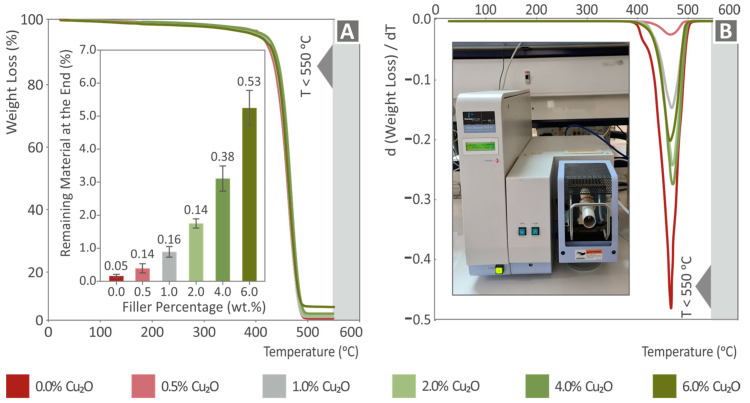
TGA analysis results: (**A**) sample weight (%) vs. temperature (°C) for all tested materials. In the inset figure the remaining mass in wt.% in each case after the completion of the measurement is presented, along with the deviation from three repetitions, and (**B**) weight loss rate (dw/dT) vs. temperature (°C) for all tested materials.

Thermogravimetric analysis was performed for pure PA12 all and its cuprous oxide nanocomposite variations. Figure 11A presents the percentile weight loss sample (%) versus the measured temperature for all tested materials. It can be validated that after the burnout of PA12 up to 550 °C, the weight of the remnants of cuprous oxide is in good agreement with the corresponding weight of the filler used for the fabrication of the nanocomposite filaments. In the inset figure of Figure 11A, the remaining mass of wt.% in each case after the completion of the measurement is presented, along with the deviation from the three repetitions. The TGA measurements were up to 550 °C but from 500 °C the remaining material mass remained constant. The values agree with the concentration of the filler. In all cases, slightly lower values than the nominal concentration were measured, and differences can be attributed to the compounding process, particularly during the milling processes and loading of the extruder, in which some Cu_2_O particles could be lost. These differences can also be attributed to the instrument’s accuracy. The curves’ rates until the critical point of weight loss are almost identical, which implies that the addition of Cu_2_O in PA12 neither deteriorates nor enhances its thermal stability at these operating temperatures. Figure 11B presents the weight loss rate versus the corresponding temperature for all tested materials, and it verifies the thermal performance described above. A maximum weight loss rate is achieved as the cuprous oxide loading increases.

### 3.4. Antibacterial Efficacy

**Figure 12:**
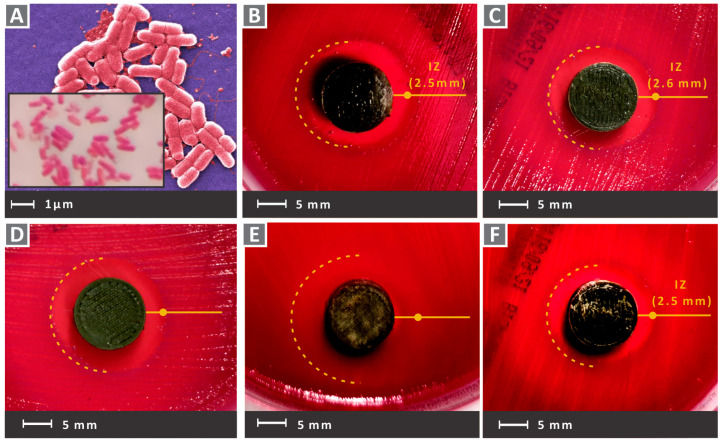
(**A**) A typical morphology of gram-negative *E. Coli* captures of the tested samples for *E. Coli* in the petri dishes after the 24 h cultivation as follows (**B**) PA12/Cu_2_O 0.5 wt.%, (**C**) PA12/Cu_2_O 1.0 wt.%, (**D**) PA12/Cu_2_O 2.0 wt.%, (**E**) PA12/Cu_2_O 4.0 wt.%, (**F**) PA12/Cu_2_O 6.0 wt.%.

**Figure 13:**
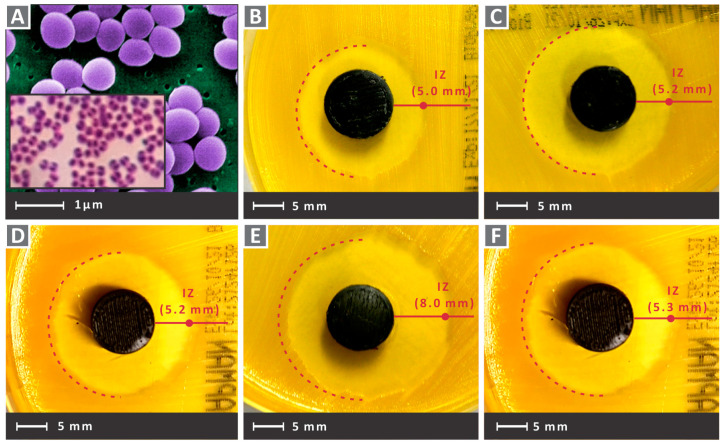
(**A**) A typical morphology of gram-positive *S. Aureus* captures of tested samples for gram-positive *S. Aureus* in Petri dishes after 24 h cultivation, as follows: (**B**) PA12/Cu_2_O 0.5 wt.%, (**C**) PA12/Cu_2_O 1.0 wt.%, (**D**) PA12/Cu_2_O 2.0 wt.%, (**E**) PA12/Cu_2_O 4.0 wt.%, and (**F**) PA12/Cu_2_O 6.0 wt.%.

In the images of Figure 12 and Figure 13, Petri dishes with gram-negative *E. Coli* and gram-positive *S. Aureus,* respectively, are presented, after the 24 h bacteria cultivation for the five PA12 cuprous oxide nanocomposites. Inhibition zones are shown for PA12/Cu_2_O 0.5 wt.% to PA12/Cu_2_O 6.0 wt.%, respectively, in Figure 12B–F and Figure 13B–F for each strain. The presence of cuprous oxide creates a rather wide inhibition zone for both tested bacteria and every filler concentration tested. A better antibacterial efficacy was observed for gram-negative *E. Coli*, while the larger inhibition zone was measured for PA12/Cu_2_O 4.0 wt.% in gram-negative *E. Coli* (Figure 12E).

**Figure 14:**
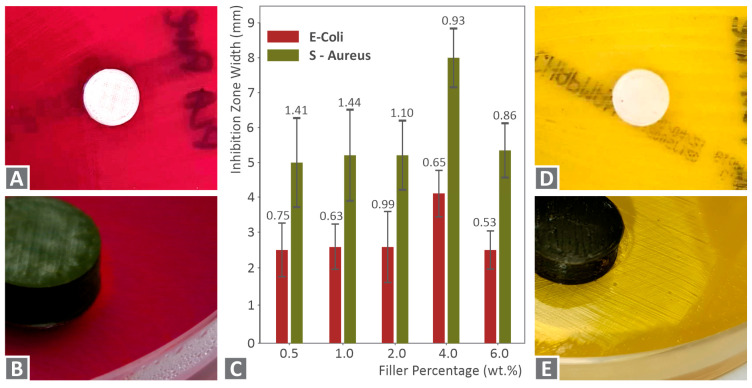
(**A**) Pure PA12 antibacterial test results against *E. Coli;* (**B**) close look at gram-negative *E. Coli* Petri dish after 24 h; (**C**) inhibition zone width (mm) measured per each bacterium and filler concentration (wt.%), along with their deviations; (**D**) Pure PA12 antibacterial test results against *S. Aureus*; (**E**) close look of gram-positive *S. Aureus* petri dish after 24 h.

In Figure 14A,D the pure PA12 antibacterial tests results for the two bacteria tested are shown. As expected, no antibacterial performance can be observed. This is a medical-grade material, which means that it is biotolerable and can be used in several medical applications. The material has to be sterilized according to medical standards. Still, there is no specification that the specific pure PA12 grade used in this work has antibacterial performance, and this was verified in the tests of this work. In Figure 14B, a closer look at a typical Petri dish after 24 h of cultivation is shown for gram-negative *E. Coli*. Figure 14C presents inhibition zone widths measured for all tested nanocomposite materials comparably for cultivations of each bacterium, along with their deviations (three measurements were taken for each case). The cuprous oxide ratio of 4.0 wt.% exhibited the highest antibacterial efficacy for both bacteria. For the other filler concentrations, the measured inhibition zones were approximately 50% narrower than the 4.0 wt.% but they still exhibited antibacterial activity. Figure 14E shows, also, a typical closer look at the Petri dish after 24 h cultivation of gram-positive *S. Aureus*.

## 4. Discussion

**Figure 15:**
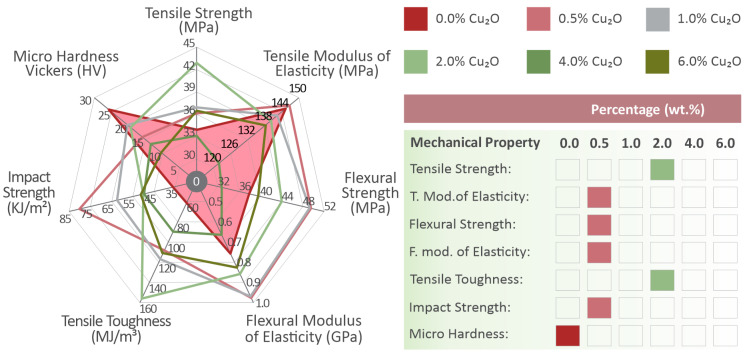
Overall results regarding the mechanical performance of PA12-Cu_2_O nanocomposite materials and pure PA12. The shaded area shows the pure material performance.

In Figure 15, a summary of the measured and calculated mechanical property values is shown in a comparable way for all tested materials. Overall, cuprous oxide in the loading of 0.5 wt.% exhibited the highest values, although the mechanical behavior of PA12 was affected by the presence of cuprous oxide at all concentrations studied. In most cases, Cu_2_O created a stiffening effect and a change in the rather elastic behavior of the pure PA12 material. With the addition of 0.5 wt.% cuprous oxide, the elastic behavior of PA12 was not significantly affected, although a slight strengthening effect occurred. These advantages are creating a potential for cuprous oxide, as the mechanical performance enhancement for PA12. Additionally, its ability to extend enough before it breaks is not affected negatively by the filler existence. At low concentrations, cuprous oxide does not also affect material flow observed optically from the 3D printer’s extrusion nozzle and the morphology of the matrix material, making the nanocomposite suitable for use in the FFF AM technology. In some measurements, the PA12 nanocomposite with 4.0 wt.% loading performed in a better way when compared to the 0.5 wt.%. For example, the calculated toughness (Figure 5A) was found to be approximately 43% higher for 4.0 wt.% when compared to the 0.5 wt.%, while the 0.5 wt.% toughness was found to be 80% higher than the pure PA12 one. As a general mechanical performance comment, one could assume that cuprous oxide slightly affects the mechanical properties of PA12, in such a way that loading increase creates a rather inelastic behavior. Low concentration PA12/Cu_2_O nanocomposites, such as 0.5 wt.%, could create a potential for a tensile strength increase in the mechanical performance when compared to the pure one, while almost no effect occurs in the material flow observed from the 3D printer’s extrusion nozzle and the material morphology. Studies with a potential for a comparison of the current study’s reported results were searched, but no similar study was found in the existing literature regarding polyamides or even polymers/cuprous oxide nanocomposites.

The roughness analysis conducted during the current study confirms the low effect of cuprous oxide in PA12. From the roughness measurements of the filament surfaces, the specimens, and the SEM images, a rather stable outcome, even at higher filler concentrations of 4.0 wt.% and 6.0 wt.%, was revealed. Some minor voids observed at 6.0 wt.% and the unsuccessful attempt of the PA12/Cu_2_O 8.0 wt.% nanocomposite could probably describe a plausible saturation at such loadings. Minor deterioration in the mechanical performance also supports this assumption. Fracture mechanisms and stiffening effects were also confirmed through the SEM analysis performed as described above, and the estimation for an optimum cuprous oxide loading of 0.5 wt.% was further strengthened through this analysis.

The thermal analysis revealed a slight increase of the thermal resistance with the increase of filler loading. This means that cuprous oxide addition to the PA12 matrix creates a more enhanced performance through the temperature increase, as not even the break-even point of high weight loss rates is increased, but also a reduction in such weight loss rate was exhibited. Thermal performance was analyzed through D/TGA, indicating that the addition of the filler does not compromise the thermal stability of the matrix material.

As for the antibacterial performance, a mild performance was reported for all studied cases, but it was shown that PA12/Cu_2_O 4.0 wt.% nanocomposite material exhibited the highest antibacterial performance for both strains tested. Similar behavior is presented from Gurianov et al. [71], in their study with a polyethylene matrix. Utilizing a different antibacterial activity test, they demonstrated that the addition of cuprous oxide in a polymer matrix could provoke an intense antibacterial efficacy. The highest mechanical response was obtained for the 0.5 wt.% filler loading, while the highest antibacterial performance was found for the 4 wt.% filler loading. In this context, given the multifunctionality of the examined nanocomposites, the selection of the filler concentration rests on the specific needs of the application to be used.

Research in the PA12 polymer for MEX applications is focusing mainly on the tensile response for the investigation of the mechanical properties [31,35,49], or in the exploitation of statistical modeling tools to optimize the 3D printing parameters [45,48]. Carbon fiber additives significantly enhanced the mechanical response of PA12 [47], while literature in PA12 with nanoparticle (NPs) additives for MEX is very limited. PA12 has been exploited in applications related to the antibacterial performance of materials, while in these cases research was not focusing on the mechanical response of the materials [52,53,54], or tensile tests only were conducted [51]. Cuprous oxide is widely used as a filler to induce antibacterial performance in composites; however, this research focused mainly on the medical parameters and not on the mechanical response of the prepared materials [61,62]. In this work, nanocomposites for MEX, AM were prepared with PA12 as the matrix material and cuprous oxide as the filler in the NP form and exhibited antibacterial performance and enhanced mechanical response when compared to the matrix material. A full set of tensile, flexural, impact, and microhardness mechanical tests were conducted to characterize the prepared materials, according to international standards.

The nanocomposites prepared in this work exhibited multi-functional performance with an improved mechanical, thermal, and antibacterial response, while at the same time being cost-efficient. The cost-efficiency is derived by the fact that the addition of the filler in the matrix material induces antibacterial performance to the prepared nanocomposite, which has superior mechanical performance when compared with the matrix material. Meanwhile, the increase in the material costs (not the total cost) is around 37% for the 2.0 wt.% nanocomposite, which demonstrated the optimum performance overall (EUR 5 vs. EUR 6.8 for 100 gr material, which is a rather small increase). This cost increase is estimated considering that 25 Kgr PA12 costs EUR 337 ex-works (for many packages), which is roughly EUR 1300 delivered for one package, which is EUR 0.05/gr for the matrix material. Cuprous oxide costs EUR 674/Kgr on the vendor’s website, which is roughly EUR 880–1000 delivered and is EUR 0.9/gr for the additive of this work. Considering the amount of the filler in the nanocomposite according to its loading, the increase in the material’s cost with the addition of the filler can be estimated for each case. Some additional preparation costs should be also considered, which are evaluated to be minimal for industrial-scale applications. In addition, these are rough estimations for laboratory-scale quantities—for industrial-scale quantities, these values would be significantly lower.

## 5. Conclusions

In the current study, multifunctional PA12/Cu_2_O nanocomposites were fabricated in the form of filament, and specimens were 3D printed with MEX to investigate their mechanical, thermal, morphological, and antibacterial properties. It was found that the introduction of cuprous oxide in the polymer matrix could positively enhance material performance in all the examined cases. Specifically, the addition of Cu_2_O in a concentration of 1.0 wt.% to 4.0 wt.% could result in mechanical performance enhancement, with parallel thermal and antibacterial property improvement when compared to the properties of pure PA12 and the materials with up to 6 wt.% fillers loading were printable using the chosen conditions.

In the case that someone would like to enhance the performance of PA12 mechanical, thermal, and antibacterial properties, without compromising the easiness of processing during the filament extrusion and the FFF 3D printing process, a golden composition of 2.0 wt.% cuprous oxide is suggested, as documented by the results of this study. Increasing the concentration of cuprous oxide addition above 4.0 wt.% would cause a saturation, driving such loading as a safe threshold point for PA12 cuprous oxide nanocomposites.

Overall, cost-efficient multi-functional materials for MEX 3D printing were prepared, characterized, and presented in this work, showing the expected improvement in the matrix material with the addition of this specific and well known for its antibacterial properties filler (Cu_2_O), in the NP form, which is widely used as an enhancement material in several applications. The advantages of 3D printing could also be exploited in applications requiring these specifications, while any processability issues were reported and discussed.

## Data Availability

The data presented in this study are available upon request from the corresponding author.
